# MPprimer: a program for reliable multiplex PCR primer design

**DOI:** 10.1186/1471-2105-11-143

**Published:** 2010-03-18

**Authors:** Zhiyong Shen, Wubin Qu, Wen Wang, Yiming Lu, Yonghong Wu, Zhifeng Li, Xingyi Hang, Xiaolei Wang, Dongsheng Zhao, Chenggang Zhang

**Affiliations:** 1 Beijing Institute of Radiation Medicine, State Key Laboratory of Proteomics, Beijing 100850, PR China; 2 Beijing Institute of Health Administration and Medical Information, Beijing 100850, PR China

## Abstract

**Background:**

Multiplex PCR, defined as the simultaneous amplification of multiple regions of a DNA template or multiple DNA templates using more than one primer set (comprising a forward primer and a reverse primer) in one tube, has been widely used in diagnostic applications of clinical and environmental microbiology studies. However, primer design for multiplex PCR is still a challenging problem and several factors need to be considered. These problems include mis-priming due to nonspecific binding to non-target DNA templates, primer dimerization, and the inability to separate and purify DNA amplicons with similar electrophoretic mobility.

**Results:**

A program named MPprimer was developed to help users for reliable multiplex PCR primer design. It employs the widely used primer design program Primer3 and the primer specificity evaluation program MFEprimer to design and evaluate the candidate primers based on genomic or transcript DNA database, followed by careful examination to avoid primer dimerization. The graph-expanding algorithm derived from the greedy algorithm was used to determine the optimal primer set combinations (PSCs) for multiplex PCR assay. In addition, MPprimer provides a virtual electrophotogram to help users choose the best PSC. The experimental validation from 2× to 5× plex PCR demonstrates the reliability of MPprimer. As another example, MPprimer is able to design the multiplex PCR primers for DMD (dystrophin gene which caused Duchenne Muscular Dystrophy), which has 79 exons, for 20×, 20×, 20×, 14×, and 5× plex PCR reactions in five tubes to detect underlying exon deletions.

**Conclusions:**

MPprimer is a valuable tool for designing specific, non-dimerizing primer set combinations with constrained amplicons size for multiplex PCR assays.

## Background

Multiplex polymerase chain reaction (PCR), defined as the simultaneous amplification of multiple regions of a DNA template or multiple DNA templates through the use of multiple primer sets (PS, comprising a forward primer and a reverse primer) in one tube, has been widely used in diagnostic applications of clinical [1,2] and environmental microbiology studies [3]. The key step in running a successful multiplex PCR reaction is to design an optimal primer set combination (PSC, a group of PSs, PSCs for primer set combinations). It is well known that for conventional PCR, the optimal PS has the following standards or properties: 1) primer size: 18-30 bp; 2) product size: 100-500 bp; 3) melting temperature (Tm) of both forward and reverse primers: 58-65°C, with a temperature difference of less than 3°C; 4) GC content of primers: 40-60%; 5) ΔG (Gibbs free energy) of the last five resides of the primers at the 3' end: ≥ -9 kcal/mol; *etc *[4,5]. However, there are several additional criteria which must be taken into account when considering multiplex PCR assay: 1) lack of primer dimerization between all of the primers; 2) similarity of the Tms of each primer; 3) primer specificity to avoid mis-priming; and 4) constraint of electrophoretic mobility of the amplicons in order to separate and purify the DNA fragments easily in agarose gel electrophoresis [6-9]. 

It has been proved by Nicodeme and Steyaert that determining minimum-set primers for multiplex PCR is an NP-complete problem [10]. Most of the current programs mainly focus on the issue of determining the minimum-set primers, such as MPP (greedy algorithm) [11], PDA-MS/UniQ (modified compact genetic algorithm) [12], G-PRIMER (greedy algorithm) [13], and Greene SCPrimer (greedy algorithm) [14]. There are also other programs available for the design of primers in specific contexts. For example, Primaclade [15] designs minimally degenerate primers for comparative studies of multiple species. Primique [16] designs PCR primers specific for each sequence in a gene family. PrimerStation [8] designs human-specific multiplex PCR primers by checking the entire human genome database. MuPlex [5,6] utilizes a multi-node graph algorithm derived from a greedy algorithm to assign and partition single nucleotide polymorphisms (SNP) into multiplex-compatible tubes for SNP genotyping. However, with the exception of PrimerStation, few of the programs mentioned above analyze the primer specificity against the genomic or transcript DNA database. Additionally, only a few of these programs provided a simple BLAST [17] search against the database locally or the GenBank database to examine the specificity of PCR primers [6,11]. For example, the only database used to check primer specificity in PrimerStation is the human genome database. Moreover, few of the primer design programs constrain the amplicon size to allow separation and purification of the DNA fragments in agarose gel electrophoresis when designing PSCs for multiplex PCR. Some programs simply define a fixed length (for example, 10 bp) as the minimum size difference between the amplicons. However, the relation between an amplicons' size and their electrophoretic mobility in agarose gel electrophoresis is absolutely nonlinear [18]. For example, it is easy to separate two DNA fragments of 100 bp and 150 bp in agarose gel (0.5%-2%) electrophoresis, but quite difficult to separate two amplicons of 1000 bp and 1050 bp in a similar gel. The electrophoretic mobility of the amplicons should be considered at the very beginning of primer design.

In this work, we developed a program named MPprimer to address these issues. It employs the widely used primer design program Primer3 [4] and the primer specificity evaluation program MFEprimer [9] to design and evaluate candidate primers based on genomic or transcript DNA databases, followed with primer dimerization examination to discard unsuitable primers. Finally, a graph-expanding algorithm derived from a greedy algorithm was used to determine the optimal PSCs for multiplex PCR. MPprimer then provides a virtual electrophotogram to help users choose the best PSCs for multiplex PCR assay.

## Implementation

**Figure 1:**
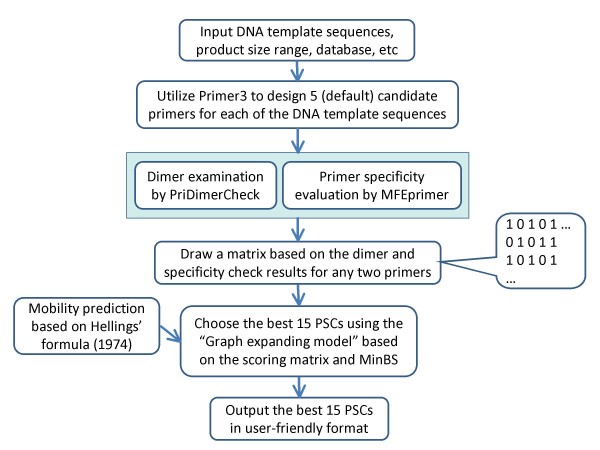
**Flowchart of MPprimer on primer design for multiplex PCR assay**.

Primer3 has been widely used for conventional PCR primer design. MPprimer utilized Primer3 to design candidate PSs for each of the DNA template sequences. To ensure that the PSs (one PS for one template sequence) in a multiplex PCR work efficiently in the same tube, the optimal parameters for the entire template sequences were used for primer design by Primer3. These parameters (and their default values) are: Tm (57-63°C), primer size (18-27 bp), GC content (45-55%), maximum 3' stability (9.0 kcal/mol), maximum self-complementarity (8.0) and maximum 3' self-complementarity (3.0). It should be noted that, instead of using the default settings in Primer3, MPprimer uses the thermodynamics parameters suggested by Santalucia [19] to calculate Gibbs free energy (ΔG) and Tm. For each template sequence, five candidate PSs were designed by Primer3 by default and ordered from a small to large penalty (the smaller the penalty, the better the PS). One PS for each template was selected to create a complete PSC for the defined template sequences. If the user inputs *k *number of template sequences, Primer3 will design 5 × *k *PSs in total and MPprimer will select *k *PSs to construct a PSC (Figure 1). In other words, a PSC contains *k *number of PSs, where each PS will specifically amplify its own target DNA sequence.

To avoid primer dimerization, MPprimer examines each pair of primers in a PSC using a dimer checking program named PriDimerCheck, which is part of the PerlPrimer source code [20], with some modifications. The most significant modification we made was the criterion to determine the complementarity degree of two primers. We calculated the stability of complementarity using a Nearest-Neighbor method using thermodynamic parameters instead of the matching score, which is used in many programs such as AutoDimer [21] and Primer3. Checking primer dimerization based on thermodynamics is more quantitative and reliable than the matching score method [21]. MPprimer uses a stringent cutoff value of -7 kcal/mol to define a dimer in PriDimerCheck.

MPprimer uses the MFEprimer program to evaluate the specificity of the primers in a PSC. In order to illustrate our specificity evaluation strategy, we used the terms T_i_P_f _and T_i_P_r _('T' for template and 'P' for primer) to represent forward and reverse primers of template *i*, and T_j_P_f _and T_j_P_r _for primers of template *j *(where T_i_P_f_, T_i_P_r_, T_j_P_f _and T_j_P_r _belong to one PSC). There are two steps of specificity examination in MPprimer. The first step is to evaluate the specificity of primer pair (PP) T_i_P_f _and T_i_P_r _[PP1] or primer pair of T_j_P_f _and T_j_P_r _[PP2]. This is similar to conventional PCR primer specificity examination. The second step is to evaluate the nonspecific cross amplification, for example, between primer pairs of T_i_P_f _and T_j_P_f _[PP3], T_i_P_r _and T_j_P_r _[PP4], T_i_P_f _and T_j_P_r _[PP5], or T_i_P_r _and T_j_P_f _[PP6]. All of these potential primer pairs are thoroughly examined by MFEprimer. If one of these six primer pairs [PP1~6] failed in the examination, the two PSs ([T_i_P_f_+T_i_P_r_] for T_i _and [T_j_P_f_+T_j_P_r_] for T_j_) are not allowed to co-occur in a PSC.

Following dimerization examination and specificity evaluation, a scoring matrix is developed based on the PSs examination results. If there is no dimerization and no nonspecific amplicons found between two PSs, a value of 1 is assigned to these two PSs, otherwise a value of 0 is set to generate the scoring matrix as shown in Figure 1.

### Choosing optimal PSCs using a graph-expanding algorithm

**Figure 2:**
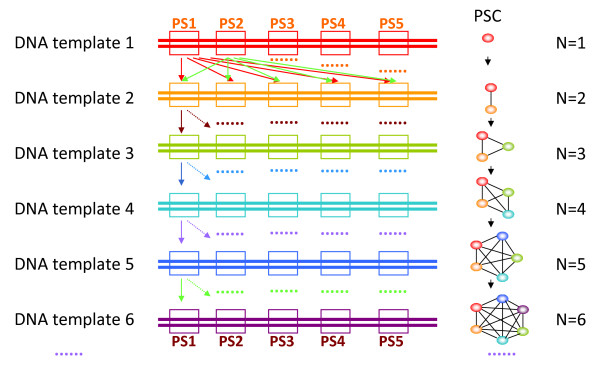
**Schematic diagram showing the construction of PSC graph with N number of primer sets**. PS: Primer set, comprising a forward and a reverse primer; PSs is the plural form of PS. **PSC**: Primer set combination, a group of PSs; PSCs is the plural form of PSC. **O**: Represents a PS, different colors represent different PSs which come from different DNA template sequences. **--**: An edge connecting two PSs indicates that they are compatible to work together in one tube without any interference or competition to produce nonspecific amplicons. **N**: Represents the number of DNA template sequences in multiplex PCR.

We used a graph-expanding algorithm derived from a greedy algorithm to choose the optimal PSC. In the graph model (Figure 2), a node represents a candidate PS and an edge represents a specific relationship between two PSs. An edge connecting two PSs indicates that they are able to work in one tube without any interference or competition to produce nonspecific amplicons. After the first two PSs (nodes) are linked together by one edge (PS1 and PS2), another node is found and added to this graph to join to two new edges (PS3 with PS1 and PS3 with PS2, Figure 2, right panel). Whether two nodes can be connected is based on the primer dimerization and specificity evaluation results, which is displayed in the scoring matrix (described above). By repeating this procedure, we can extend the graph to give it sufficient nodes to create a candidate PSC (Figure 2). Because the PSs designed by Primer3 are sorted by the penalty values (PS1-5 in Figure 2), our graph-expanding strategy changed from randomly selected nodes [5] to the nodes with lowest penalty. Thus, the program finds the optimal PSC, as opposed to choosing a random one, without requiring additional PSCs sorting time. The scoring matrix makes sure that the PSs in a PSC are compatible. For N × 5 nodes (N× plex PCR primer design or N template sequences, 5 for candidate PSs) in the graph, pre-computing and storing the scoring matrix would require O(N^2^) time and space.

When searching PSCs, a constraint condition called MinBS (minimum band spacing) has to be satisfied. This artificial condition is defined as the minimum electrophoretic migration distance of the amplicons in 1% agarose gel electrophoresis and should be greater than a certain distance so that the DNA bands can be easily distinguished by the naked eye for purification.

Where *position*_*i *_and *position*_*j *_represents the electrophoretic mobility of the two DNA amplicons (*i *and *j*) amplified by the PSC. The formula used to predict the electrophoretic mobility of amplicons was reported by Helling *et al*., 1974 [18].

## Results and discussion

We provide both a web application and a stand-alone version of MPprimer for different purposes. The MPprimer web application is conveniently designed, with a friendly interface, while the stand-alone version is applicable to high-throughput multiplex PCR primer design with supporting comprehensive, custom-built databases in order to meet specific user demands. After inputting a set of template DNA sequences in FASTA format, MPprimer will design and output the best 15 PSCs by default in a user-friendly format. Besides the binding pattern of the primers and their template sequences, the detailed specificity evaluation results of MFEprimer are also provided. In addition, for the PSs of each PSC, users can then re-run the sequences on PriDimerCheck and MFEprimer to view the results of possible primer dimerization or nonspecific amplifications with stricter NCBI BLAST parameters such as using a lower word size (-W 4) and larger E value (-e 10000) to improve the sensitivity. Notably, MPprimer also provides a function to generate the virtual agarose gel electrophotogram for each PSC to give users a visual impression before running the real multiplex PCR reaction (see homepage of MPprimer).

Because the purpose of MPprimer is to design an optimal PSC that can specifically amplify the target DNA sequences without nonspecific amplification or primer dimerization, it is useful for designing primers for DNA template sequences without high sequence similarity. However, MPprimer does provide an alternative way to design primers for highly similar sequences (for example, several genes of one family or different transcript variants of the same gene) by allowing users to specify the region of primer location for each DNA template sequence. This requires users to first run a multiple alignment for the sequences to find the nonconserved region(s) before designing primers using MPprimer.

**Figure 3:**
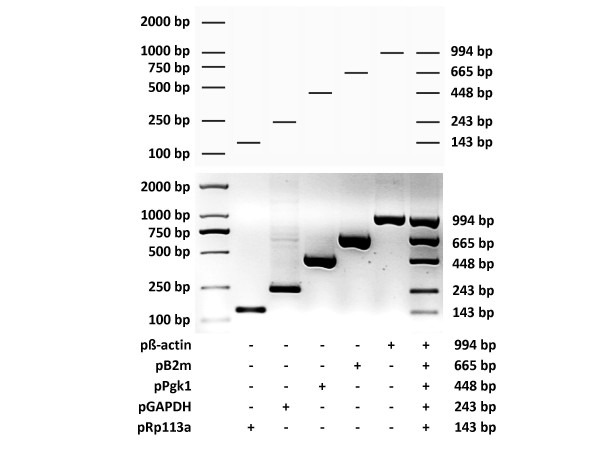
**Diagram showing the experimental validation of the multiplex PCR by using the primer set combination (PSC) designed by MPprimer for five mouse genes (β-actin, B2m, Pgk1, GAPDH and Rp113a)**. The top panel showing the predicted (virtual) electrophoretogram of five conventional PCRs and a multiplex PCR by MPprimer. The middle and bottom panels showing the experimentally validated electrophoretogram. The plus sign indicates the use of the primer set.

**Figure 4:**
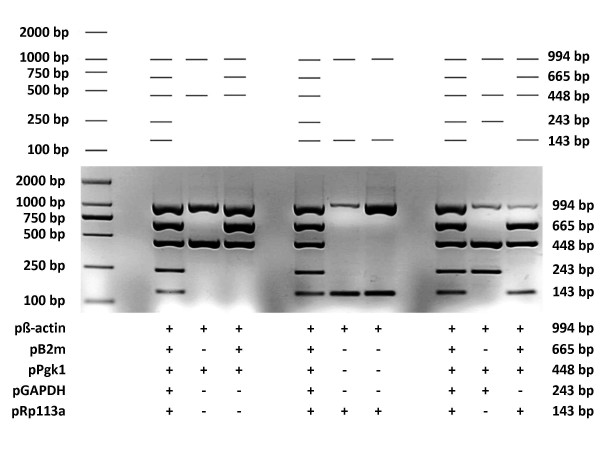
**Diagram showing the three letters "P", "C" and "R" by experimentally validated electrophoretogram using the primer set combination (PSC) designed by MPprimer for five mouse genes (β-actin, B2m, Pgk1, GAPDH and Rp113a)**. The three letters "P", "C" and "R" were shown in both a virtual electrophoretogram predicted by MPprimer (top panel) and the experimentally validated electrophoretogram (middle and bottom panels). The plus sign indicates the use of the primer set.

To examine the performance of MPprimer, we designed a PSC comprising 5 PSs (10 primers) to amplify five mouse genes (β-actin, B2m, Pgk1, GAPDH, Rpl13a) in one tube as a typical multiplex PCR reaction. The result of the real experiment was nearly the same as that predicted by the virtual agarose gel electrophoresis analysis (Figure 3 and Figure 4). The homepage of MPprimer describes the detailed information of the experiment including the templates sequences, primer sequences, and the PCR and electrophoretic conditions. As another example, we have successfully used MPprimer to design the multiplex PCR primers for DMD (dystrophin gene which caused Duchenne Muscular Dystrophy), which has 79 exons, for 20×, 20×, 20×, 14×, and 5× plex PCR reactions in five tubes to detect underlying exon deletions (see homepage of MPprimer for details).

Although designing minimum-set primers can save cost and time by reducing primer synthesis demand [11-14], it is crucial to design specific PSs, especially at the genomic or transcript level, to perform multiplex PCR with high reliability [9]. MPP [11], PDA-MS/UniQ [12], G-PRIMER [13] and Greene SCPrimer [14] are mainly concerned with the problem of minimum-set primers. PrimerStation [8] designs specific multiplex PCR primers only by checking the entire human genome database, but this database is too limited. The MPprimer web application supports transcript level specificity evaluation for more than ten species, while the stand-alone version can support any DNA sequence database, even the large genomic DNA database. MuPlex [5,6] and MPP [11] simply use BLAST [17] to check primer specificity, but this is insufficient. Moreover, several other conditions such as Tm were not considered [9,22]. Primaclade [15] and Greene SCPrimer [14] are used for degenerate primer design, while Primique [16] focuses on designing specific PCR primers for each sequence in a gene family. However, none of these programs provide the function for predicting the electrophoretic mobility of the amplicons from multiplex PCR reaction [18]. MuPlex [5,6] and MPprimer use a very similar algorithm to find PSC in a graph where nodes are PSs and edges connect compatible pairwise PSs for multiplex PCR. The difference between them is that MPprimer selects nodes which have a lower penalty (indicating higher quality [4]) rather than random ones. Therefore, MPprimer can find the optimal PSC without enumerating and sorting all the PSCs to find the optimal one. It should be noted that, as our graph-expanding model is based on the preselected candidate primer sets (MPprimer utilizes Primer3 to design 5 primer sets for each of the template sequences), the output PSCs are not global but only local optimal. In another aspect, the running time of MPprimer is incomparable to other programs, because the specificity examination by MFEprimer requires more time for sequence similarity analysis between the primer sequence and the genomic or complementary DNA database of the same species. Therefore, the MPprimer web application currently only supports transcript level specificity examination. However, the stand-alone version of MPprimer supports unlimited databases, such as the genomic DNA database, which mainly depend on the user's computing capability.

Our further plans are to: 1) automatically analyze and design primers for amplifying different transcript variants for alternative splicing analysis of a single gene; 2) provide an alternative solution, should MPprimer not find suitable primers for a set of template sequences in one tube, by suggesting two or more tubes for one PCR reaction.

## Conclusions

We developed a new program named MPprimer with both a web application and a stand-alone version to help users design highly reliable PSs for multiplex PCR assay. The web application is easy-to-use with a friendly interface, while the stand-alone version is applicable to high-throughput multiplex PCR primer design with the support of comprehensive custom-built DNA sequence databases. With the help of MPprimer, users can design reliable primer set combinations for multiplex PCR analysis.

## Availability and requirements

• **Project name: **MPprimer

• **Project home page: **http://biocompute.bmi.ac.cn/MPprimer/

• **Operating system(s): **The web application is platform independent and the stand-alone version runs on Linux/Unix.

• **Programming language: **Python

• **Other requirements: **Python ≥ 2.5

• **License: **GNU GPL v 3

• **Any restrictions to use by non-academics: **License needed

## Authors' contributions

ZS and WQ wrote the main manuscript and did the most of the programming. WW carried out the multiplex PCR experiment. YL helped to optimize the algorithm. YW analyzed the agarose gel electrophoresis to determine the electrophoretic mobility of the amplicons. ZL and XH participated in programming and debugging. XW and DZ implemented the web server construction. CZ designed and supervised the study, finalized the manuscript. All authors read and approved the manuscript.
